# Association of Gla-Rich Protein (GRP) with Inflammatory Markers in Critically Ill Patients: A Cross-Sectional Observational Study

**DOI:** 10.3390/metabo15090611

**Published:** 2025-09-13

**Authors:** Elif Eygi, Sinem Bayrakçı, Onur Bayrakçı, Nazire Ates Ayhan, Ahmet Atlas, Metin Kilinc, Recep Dokuyucu

**Affiliations:** 1 Department of Anesthesiology and Reanimation, Gaziantep City Hospital, Gaziantep 27100, Turkey; 2 Department of Intensive Care, Gaziantep City Hospital, Gaziantep 27100, Turkey; drsinembayrakci@gmail.com; 3 Department of Thoracic Surgery, Gaziantep City Hospital, Gaziantep 27100, Turkey; dronurbayrakci@gmail.com; 4 Department of Intensive Care, Şanlıurfa Training and Research Hospital, Şanlıurfa 63040, Turkey; nazireates@yahoo.com; 5 Department of Anesthesiology and Reanimation, Faculty of Medicine, Harran University, Şanlıurfa 63040, Turkey; ahmetatlas@harran.edu.tr; 6 Department of Anesthesiology and Reanimation, Faculty of Medicine, Mardin Artuklu University, Mardin 47200, Turkey; metinkilinc@artuklu.edu.tr; 7 Department of Physiology, Medical Specialization Training Center (TUSMER), Ankara 06230, Turkey; drecepfatih@gmail.com

**Keywords:** Gla-rich protein (GRP), total thiol oxidation reduction ratio (TORR), inflammation, oxidative stress, calcium metabolism, intensive care

## Abstract

Objectives: Gla-rich protein (GRP), a vitamin K-dependent protein, has been increasingly recognized for its dual role in modulating inflammation and inhibiting pathological calcification. Despite its emerging importance in chronic conditions, limited evidence exists regarding its behavior during acute critical illness. This study aimed to investigate the association between GRP, systemic inflammatory markers, oxidative stress (via total thiol oxidation-reduction ratio, TORR), and calcium metabolism in critically ill patients. Materials and Methods: This cross-sectional observational study included 93 critically ill patients admitted to the intensive care unit (ICU) and 60 age- and sex-matched non-critically ill volunteers. Serum GRP levels were measured using ELISA. Other biomarkers including TORR, C-reactive protein (CRP), procalcitonin (PCT), white blood cell count (WBC), immature granulocytes (IGs), and serum calcium were also analyzed. Pearson’s correlation, multivariate linear regression, and ROC analysis were performed to assess the relationships among GRP and biochemical markers, as well as their capacity to differentiate ICU patients from controls. Results: GRP, TORR, CRP, PCT, WBC, IGs, and ferritin levels were significantly elevated in ICU patients compared to the control group, whereas serum calcium levels were markedly reduced (all *p* < 0.05). GRP levels demonstrated moderate positive correlations with WBC (r = 0.47), neutrophils (r = 0.51), TORR (r = 0.42), CRP (r = 0.30), and IGs (r = 0.46), and a strong negative correlation with calcium (r = −0.63). In multivariate regression, TORR, CRP, WBC, IGs, PCT, and calcium levels showed significant correlations with GRP levels in univariate analysis. ROC analysis revealed that CRP had the highest discriminatory power (AUC = 0.88; 95% CI: 0.82–0.94), followed by TORR (AUC = 0.79; 95% CI: 0.71–0.86), GRP (AUC = 0.76; 95% CI: 0.68–0.84), and IGs (AUC = 0.77; 95% CI: 0.69–0.85), for distinguishing ICU patients from non-critically ill individuals. Conclusions: Our findings demonstrated that GRP is significantly associated with systemic inflammation, oxidative stress, and calcium metabolism disturbances in critically ill patients. The combined evaluation of GRP and TORR may enhance the understanding of inflammatory and oxidative mechanisms in acute critical illness. Although this study did not assess patient outcomes, these biomarkers could serve as promising candidates for future prognostic research in ICU settings.

## 1. Introduction

Chronic inflammatory diseases (CIDs) are among the leading causes of death worldwide and have been identified by the World Health Organization as one of the greatest threats to human health [1,2]. Chronic inflammation contributes to the pathophysiology of various diseases, including cardiovascular diseases (CVD), chronic kidney disease (CKD), arthritis, osteoarthritis (OA), diabetes, neurodegenerative disorders, and cancer [3,4,5,6]. One of the common features of these conditions is their frequent association with the need for intensive care admission. While chronic inflammation initially manifests as a low-grade inflammatory state, it can progress to severe and acute proinflammatory reactions leading to multiple organ dysfunction in later stages [3,6]. The lack of effective therapies for these diseases has led to the widespread use of anti-inflammatory drugs to reduce inflammation and alleviate symptoms. However, despite the availability of many anti-inflammatory agents, there remains an urgent need for new, safe, and more effective therapeutic options for the prevention and treatment of inflammation [1,7,8].

Gla-rich protein (GRP), also known as upper zone of growth plate and cartilage matrix associated protein (UCMA), is a vitamin K-dependent protein (VKDP) that plays significant roles in several processes related to the development of CIDs such as CVD, OA, rheumatoid arthritis, and CKD [9,10,11,12,13]. Recently, GRP has been proposed as a potential biomarker for vascular and valvular calcification as well as renal dysfunction [9,12,14,15]. GRP is a potent inhibitor of pathological calcification at both the tissue and systemic levels [11,16]. While γ-carboxylation of GRP is crucial for its calcification-inhibitory function, its anti-inflammatory activity has been shown to be independent of its γ-carboxylation status [17,18]. Elevated GRP levels have been associated with tissue-protective effects, whereas GRP deficiency has been linked to various disease states [9,13,19].

Although previous studies have investigated GRP in the context of chronic diseases such as CKD, cardiovascular disease, and osteoarthritis, little is known about its behavior during acute systemic inflammation and organ dysfunction in the ICU setting. Importantly, no previous study has jointly assessed GRP and TORR in critically ill patients, or their association with key inflammatory and calcium-related biochemical markers. Therefore, this study provides novel insight into the inflammatory and oxidative landscape of acute critical illness by evaluating the potential role of GRP in relation to both inflammation and mineral metabolism.

Based on this gap in the literature, the aim of this study was to evaluate the associations between serum GRP levels, inflammatory biomarkers (CRP, PCT, WBC, IGs), oxidative stress marker TORR, and calcium levels in critically ill patients admitted to the ICU. We also assessed the discriminative power of these markers to differentiate ICU patients from non-critically ill individuals using ROC analysis.

## 2. Materials and Methods

### 2.1. Study Design

This prospective, observational study was conducted at City Hospital between January 2024 and January 2025. Informed consent was obtained from all participants prior to their inclusion in the study. A total of 93 patients admitted to the intensive care unit (ICU) were enrolled. Patients were included if they were ≥18 years old and met the admission criteria of the Society of Critical Care Medicine (SCCM) [20], which include the need for organ support (e.g., mechanical ventilation, vasopressors) due to conditions such as acute respiratory failure, septic shock, postoperative complications, or multiorgan dysfunction. We also clarified the exclusion criteria, including malignancy, orthopedic trauma, pre-hospital antioxidant use, and expected ICU stay of less than 24 h. Blood samples were collected from the patients at the time of admission to the ICU, before the initiation of any treatment.

**Figure 1:**
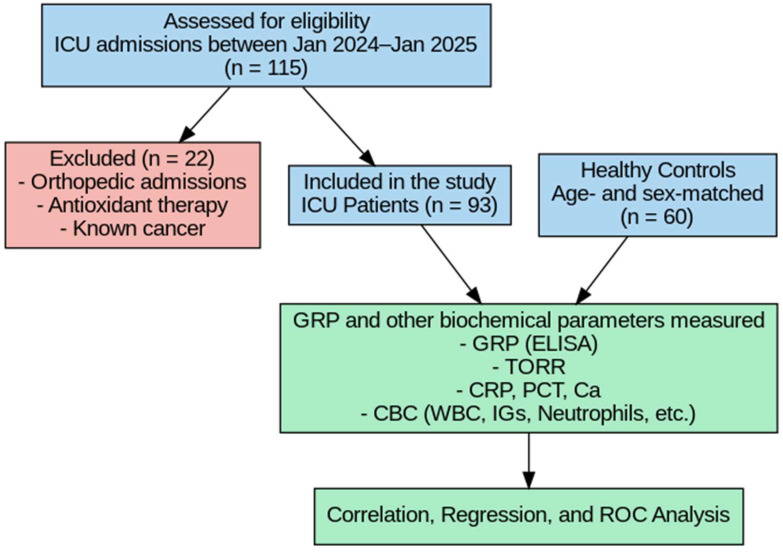
Flowchart of the study.

A control group consisting of 60 non-critically ill volunteers, matched for age and sex with the patient group at the group level (frequency matching), was also included in the study. Since each ICU patient was not individually paired with a specific control, conditional logistic regression was not required. Therefore, independent statistical methods (independent-samples tests, correlation analysis, multivariate linear regression, and ROC analysis) were applied instead of paired analyses. Although individuals in the control group did not have acute illness or require hospitalization, several had underlying chronic conditions such as diabetes or hypertension. Therefore, they were referred to as non-critically ill volunteers rather than strictly healthy individuals. Exclusion criteria were as follows: patients receiving antioxidant therapy prior to admission, those admitted to the ICU due to orthopedic conditions, and patients with a known diagnosis of cancer (Figure 1). The clinical diagnoses or etiologies leading to ICU admission (e.g., sepsis, trauma, cardiovascular events) were not categorized, and standardized clinical severity scores (such as SOFA or APACHE II) were not recorded in this study.

### 2.2. Data Collection

Clinical comorbidities such as diabetes mellitus (DM), hypertension (HTN), and chronic kidney disease (CKD) were identified based on electronic health records and prior medical diagnoses. DM was defined as a documented diagnosis or HbA1c ≥ 6.5%. HTN was defined as a history of hypertension or persistent blood pressure ≥ 140/90 mmHg. CKD was identified by an eGFR < 60 mL/min/1.73 m^2^ for more than 3 months or a prior diagnosis by a nephrologist.

### 2.3. Laboratory Parameters

Total and native thiol concentrations in both patient and control groups were automatically analyzed using a Roche Cobas 6000 c analyzer (Roche Diagnostics, Mannheim, Germany), following the manufacturer’s instructions (Rel Assay Diagnostics, Gaziantep, Turkey). The measurements were based on the “Modified Ellman Method” described by Erel et al. [8]. All results were expressed in micromoles per liter (μmol/L). After determining total thiol (-SH + -S-S-) and native thiol (-SH) levels, the following parameters were calculated:

- Disulfide level (-S-S-) = (Total thiol − Native thiol)/2
- Disulfide/Total Thiol Ratio (%) = [(-S-S-)/(Total thiol)] × 100
- Native Thiol/Total Thiol Ratio (%) = [(-SH)/(Total thiol)] × 100
- Disulfide/Native Thiol Ratio (%) = [(-S-S-)/(-SH)] × 100
- Thiol Oxidation-Reduction Ratio (TORR, %) = [Disulfide/Native thiol] × 100.

TORR was calculated using the formula [Disulfide/Native thiol] × 100, as described in prior literature. This index reflects the oxidative shift in thiol-disulfide homeostasis by quantifying the relative conversion of native thiols to disulfides. Notably, values exceeding 100% may occur when native thiol levels are substantially reduced, indicating a state of pronounced oxidative stress. However, as this calculation primarily reflects thiol oxidation, without direct measurement of reducing agents (e.g., glutathione or NADPH), it does not fully represent the dynamic redox balance. Therefore, TORR should be interpreted as an oxidative index rather than a complete redox ratio.

Procalcitonin (PCT), ferritin, C-reactive protein (CRP), white blood cell count (WBC), and immature granulocytes (IGs) were automatically analyzed using the Roche Cobas 6000 c analyzer (Roche Diagnostics, Mannheim, Germany).

### 2.4. Measurements of Gla-Rich Protein (GRP)

Serum Gla-Rich Protein (GRP) concentrations were measured using a commercially available enzyme-linked immunosorbent assay (ELISA) according to the manufacturer’s instructions. Blood samples were collected in plain tubes and allowed to clot at room temperature for 30 min, followed by centrifugation at 3000 rpm for 10 min. The obtained serum samples were aliquoted and stored at −80 °C until analysis to prevent protein degradation. Prior to analysis, all reagents and serum samples were brought to room temperature. Standards and samples were added in duplicate to 96-well microplates pre-coated with anti-GRP antibodies. Following incubation with biotin-labeled detection antibodies and streptavidin-HRP conjugate, tetramethylbenzidine (TMB) substrate solution was added to initiate the colorimetric reaction. The reaction was stopped with sulfuric acid, and the absorbance was read at 450 nm using a microplate reader. GRP concentrations were calculated from the standard curve generated using known concentrations of recombinant GRP. The sensitivity, intra-assay, and inter-assay coefficients of variation (CV) of the assay were within acceptable limits as defined by the kit manufacturer.

### 2.5. Statistical Analysis

All statistical analyses were performed using SPSS version 27.0 (IBM Corp., Armonk, NY, USA). The normality of data distribution was assessed using the Kolmogorov–Smirnov test. Continuous variables are presented as mean ± standard deviation (SD), and categorical variables as counts and percentages. Comparisons between patient and control groups were made using the independent samples t-test for continuous variables and the chi-square test for categorical variables. Pearson’s correlation analysis was used to evaluate the relationships between Gla-rich protein (GRP), Total Thiol Oxidation Reduction Ratio (TORR), and other laboratory parameters in ICU patients. The strength of correlations was interpreted as follows: r < 0.2 (very weak or no correlation), 0.2–0.4 (weak correlation), 0.4–0.6 (moderate correlation), 0.6–0.8 (high correlation), and > 0.8 (very high correlation). To identify independent predictors of GRP levels, multivariate linear regression analysis was conducted including variables that were significant in univariate analyses. Multicollinearity was assessed using the Variance Inflation Factor (VIF), and variables with VIF > 5 were excluded. All final predictors had VIF < 2. Receiver Operating Characteristic (ROC) curve analysis was performed to determine the diagnostic performance of GRP, TORR, CRP, WBC, IGs, PCT, and calcium (Ca) levels in differentiating ICU patients from non-critically ill volunteers. The area under the curve (AUC), optimal cut-off values, sensitivity, specificity, and 95% confidence intervals (CIs) were reported. A *p*-value < 0.05 was considered statistically significant.

## 3. Results

Comparison of sociodemographic and laboratory parameters between ICU patients and non-critically ill controls was shown in Table 1. A total of 93 patients admitted to the intensive care unit (ICU) and 60 non-critically ill volunteers were included in the study. The mean age of the patient group was higher than that of the control group, but the difference was not statistically significant (63.1 ± 20.0 vs. 59.2 ± 21.4 years; *p* = 0.190). There was no significant difference in gender distribution between the groups (*p* = 0.260). However, the prevalence of comorbidities including diabetes mellitus (35.5% vs. 8.3%; *p* < 0.001), hypertension (41.9% vs. 10%; *p* < 0.001), cardiovascular disease (48.4% vs. 11.7%; *p* < 0.001), chronic kidney disease (29% vs. 5%; *p* < 0.001), and respiratory system disease (21.5% vs. 3.3%; *p* < 0.001) was significantly higher in ICU patients compared to controls. Laboratory parameters revealed significantly elevated levels of WBC, neutrophils, monocytes, mean platelet volume (MPV), ferritin, procalcitonin (PCT), immature granulocytes (IGs), Gla-rich protein (GRP), TORR, and CRP in ICU patients compared to controls (all *p* < 0.05). Conversely, lymphocyte count, red blood cells (RBC), hemoglobin (Hgb), and serum calcium (Ca) levels were significantly lower in the patient group (all *p* < 0.05) (Table 1).

Correlation Analysis of GRP, TORR, and Biochemical Parameters in ICU Patients is shown in Table 2. In correlation analysis among ICU patients, GRP levels demonstrated a moderate positive correlation with WBC (r = 0.47, *p* = 0.001), neutrophil count (r = 0.51, *p* = 0.001), monocytes (r = 0.29, *p* = 0.003), MPV (r = 0.25, *p* = 0.003), ferritin (r = 0.30, *p* = 0.002), PCT (r = 0.34, *p* = 0.001), and IGs (r = 0.46, *p* = 0.001). In contrast, GRP showed a moderate negative correlation with lymphocyte count (r = −0.31, *p* = 0.002) and a strong negative correlation with serum calcium levels (r = −0.63, *p* = 0.001). Similarly, TORR exhibited moderate positive correlations with WBC (r = 0.48, *p* = 0.010), neutrophil count (r = 0.44, *p* = 0.020), monocytes (r = 0.27, *p* = 0.004), MPV (r = 0.26, *p* = 0.002), ferritin (r = 0.33, *p* = 0.001), PCT (r = 0.36, *p* = 0.001), and IGs (r = 0.49, *p* = 0.010). Like GRP, TORR also demonstrated a significant negative correlation with calcium (r = −0.65, *p* = 0.001) and lymphocytes (r = −0.30, *p* = 0.001) (Table 2).

Linear regression analysis results for patients in the ICU were shown in Table 3. To ensure the validity of the regression model, we calculated the variance inflation factor (VIF) for each variable. All included variables had VIF values < 2, confirming that multicollinearity was not a significant issue. In multivariate linear regression analysis, GRP levels were independently associated with several laboratory parameters. GRP demonstrated a strong positive association with TORR (β = 0.42, *p* = 0.001), CRP (β = 0.30, *p* = 0.003), WBC (β = 0.28, *p* = 0.005), IGs (β = 0.29, *p* = 0.006), and PCT (β = 0.26, *p* = 0.008). Conversely, calcium levels showed a significant negative association with GRP (β = −0.35, *p* = 0.001) (Table 3).

**Figure 2:**
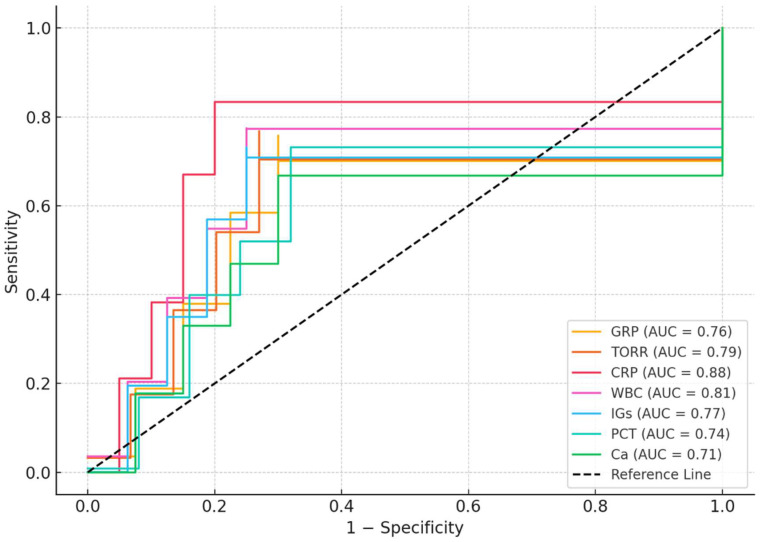
ROC analysis results for patients in the ICU.

ROC analysis results for patients in the ICU were shown in Table 4. ROC analysis demonstrated that CRP had the highest diagnostic performance for distinguishing ICU patients from controls (AUC = 0.88, 95% CI: 0.82–0.94; cut-off > 30.0 mg/L; sensitivity: 85%, specificity: 80%; *p* < 0.001). Among the other parameters, WBC (AUC = 0.81), TORR (AUC = 0.79), IGs (AUC = 0.77), GRP (AUC = 0.76), PCT (AUC = 0.74), and calcium (AUC = 0.71) also showed significant predictive value (all *p* < 0.01). The optimal cut-off values for GRP and TORR were >4.0 ng/mL (sensitivity: 72%, specificity: 70%) and >160% (sensitivity: 75%, specificity: 73%), respectively (Table 4, Figure 2).

## 4. Discussion

In this cross-sectional observational study, we investigated the relationship between GRP, inflammatory markers, oxidative stress parameters, and calcium homeostasis in critically ill patients admitted to the ICU. Our findings demonstrate that GRP levels were significantly higher in ICU patients compared to non-critically ill volunteers and were strongly associated with several inflammatory and oxidative stress markers. Additionally, GRP showed a significant negative correlation with serum calcium levels, suggesting a potential interplay between GRP, calcium metabolism, and systemic inflammation in critically ill patients.

The role of GRP in chronic inflammatory and metabolic disorders has been reported in some studies in the literature [9,11]. Silva et al. demonstrated that elevated GRP levels may serve as an early biomarker for vascular calcification and renal dysfunction, particularly in diabetic patients with chronic kidney disease (CKD) [9]. Marreiros et al. reported that GRP levels correlate with vascular calcification, inflammation, and mineral metabolism markers in peritoneal dialysis patients, highlighting its potential role in the pathophysiology of chronic diseases with inflammatory components [11]. In our study, we observed higher GRP levels in our ICU cohort, which included a high proportion of patients with comorbid conditions such as diabetes mellitus, CKD, and cardiovascular disease. Although many ICU patients have underlying chronic diseases, the inflammatory and oxidative responses observed in critical illness often represent distinct and more severe pathophysiological mechanisms.

In the present study, GRP levels were positively correlated with inflammatory markers such as WBC, neutrophils, ferritin, CRP, PCT, and immature granulocytes, and oxidative stress indicators such as TORR. This is in agreement with the findings of Viegas et al., who suggested that GRP may act as both a calcification inhibitor and an immunomodulator in the context of vascular smooth muscle cell dysfunction and chronic inflammation [19]. Xiao et al. emphasized the dual role of GRP in calcification and inflammation, suggesting that GRP may modulate inflammatory pathways independently of its γ-carboxylation status [10].

Another important finding of our study was the strong negative correlation between GRP and calcium levels. Zengwei et al. reported an inverse relationship between GRP and calcium homeostasis in patients with coronary artery calcification, suggesting that GRP may be upregulated as a compensatory response to limit calcium deposition in vascular tissues during inflammatory states [16]. In our study, although a strong inverse correlation was observed between GRP and total serum calcium levels (r = −0.63), this finding should be interpreted with caution. Total calcium does not accurately reflect tissue-level calcification or calcium bioavailability, particularly in critically ill patients where hypocalcemia may arise from hypoalbuminemia, renal dysfunction, systemic inflammation, or redistribution phenomena. Furthermore, ionized calcium—which more precisely represents physiologically active calcium—was not measured in our study. Therefore, the observed GRP–calcium association may reflect indirect or compensatory mechanisms rather than a direct causal relationship. Future studies incorporating ionized calcium measurements and calcification imaging modalities (e.g., vascular calcium scoring) are warranted to clarify this relationship.

The significant correlations between TORR and both GRP and inflammatory markers observed in our study highlighted the role of oxidative stress in the pathogenesis of critical illness. Polat et al. demonstrated that patients with rheumatoid arthritis exhibited significant impairment in thiol/disulfide balance, characterized by elevated disulfide levels and decreased native and total thiol levels, which were significantly correlated with disease activity scores (DAS28) [21]. Efiong et al. highlighted that GRP not only serves as a potent inhibitor of vascular calcification but also exhibits immunomodulatory and anti-inflammatory properties, particularly in the context of diabetic nephropathy, where inflammation, oxidative stress, and disrupted calcium-phosphate homeostasis drive disease progression [22]. Sebel et al. previously demonstrated that thiol-disulfide homeostasis is disrupted in patients with chronic inflammatory diseases such as rheumatoid arthritis, which supports our findings of elevated TORR levels in ICU patients [23]. Furthermore, Furman et al. and Chen et al. emphasized that oxidative stress and chronic inflammation are tightly interconnected, contributing to the progression of various critical conditions [1,3].

Our regression analysis revealed that TORR, CRP, WBC, IGs, PCT, and calcium levels showed significant correlations with GRP levels in univariate analysis. indicating that GRP reflects both inflammatory burden and oxidative imbalance in critically ill patients. ROC analysis further demonstrated that GRP and TORR possess moderate predictive value for distinguishing ICU patients from non-critically ill volunteers, although CRP remained the most sensitive and specific marker. This suggested that while traditional inflammatory markers remain valuable, GRP and TORR may offer additional pathophysiological insights and potential clinical utility in monitoring inflammation and oxidative stress status in ICU patients. Although TORR values exceeded 100% in our results, this was due to significantly reduced native thiol concentrations in ICU patients, which increases the disulfide/native thiol ratio disproportionately. This finding reflects a dominant oxidative state rather than an absolute disulfide excess. Nonetheless, interpretation of TORR values > 100% requires cautious clinical correlation, and further studies are warranted to validate this metric against established oxidative stress biomarkers. Since some inflammatory markers (e.g., WBC, neutrophils, IGs) were highly correlated, we assessed multicollinearity using VIF. By excluding variables with VIF > 5, we minimized collinearity bias and retained the most clinically relevant predictors. Although we considered the use of principal component analysis to consolidate correlated markers, this was not applied due to the sample size and the desire to maintain clinical interpretability. Future studies with larger cohorts may benefit from dimensionality reduction techniques to further optimize model stability.

Importantly, to our knowledge, few studies in the literature have simultaneously examined the relationship between GRP, oxidative stress parameters, and calcium metabolism in the setting of critical illness [24,25,26,27]. Our study provided novel data that may contribute to a better understanding of the complex interactions between these systems and their potential relevance to the management and prognosis of critically ill patients.

Another important consideration is the role of hypocalcemia in critical illness and its potential interplay with GRP. Hypocalcemia is frequently observed in ICU patients and may result from diverse mechanisms, including decreased parathyroid hormone secretion, intracellular sequestration of calcium, redistribution into third spaces such as ascitic fluid, and hypomagnesemia, especially in septic states. While our study demonstrated a significant negative correlation between serum calcium and GRP levels, it remains unclear whether GRP plays an active role in calcium regulation during acute inflammation, or whether its elevation reflects underlying chronic disorders associated with altered mineral metabolism. Importantly, we did not assess ionized calcium levels or sepsis-specific dynamics, which limits causal interpretation. Future studies with stratification based on disease severity and dynamic measurement of ionized calcium and GRP over time may clarify this relationship.

While our findings demonstrated significant associations between GRP, TORR, and inflammatory markers, it is important to note that the study did not assess clinical endpoints such as mortality, length of ICU stay, or progression to organ failure. Given that GRP levels can be elevated in both chronic conditions and acute critical illness, establishing specific threshold values that distinguish acute-phase elevations from chronic disease–related changes is crucial for clinical application. Our study was not designed to determine such cut-off levels; this remains a priority for future multicenter investigations involving stratified patient populations. Furthermore, future longitudinal studies are warranted to determine whether GRP and TORR can serve as predictive biomarkers for adverse outcomes in critically ill patients and thereby guide clinical decision-making.

### Limitations of the Study

This study has certain limitations. First, the sample size was relatively limited, and the single-center design may restrict the generalizability of our findings. Second, we did not perform longitudinal follow-up to evaluate the dynamic changes of GRP and TORR during ICU stay or their association with clinical outcomes such as mortality or organ dysfunction. Additionally, we did not evaluate patient outcomes such as mortality or organ failure, which limits the ability to assess the prognostic utility of GRP and TORR in clinical practice. In addition, the clinical etiologies of ICU admission were not categorized, and we did not assess disease severity using standard ICU scoring systems such as SOFA or APACHE II. This limits our ability to analyze the relationship between biomarker levels and the severity or type of critical illness. Moreover, we did not evaluate ionized calcium levels or differentiate between causes of hypocalcemia, such as sepsis, hypomagnesemia, or ascites. These unmeasured confounding factors may affect the observed relationship between GRP and total calcium. **Furthermore, while age and sex were matched at the group level, the sample size of the control group (n = 60) was smaller than the ICU group (n = 93), which may limit statistical power. However, this size was deemed adequate based on a priori power calculation assuming an effect size of d = 0.6, α = 0.05, and power = 0.80.** Further multicenter, prospective studies with larger cohorts and long-term follow-up are needed to validate our findings and clarify the potential prognostic role of GRP and TORR in critical illness.

## 5. Conclusions

In conclusion, our study demonstrated the complex interplay between inflammatory processes, oxidative stress, and calcium metabolism by evaluating multiple biochemical parameters. The findings underscored the clinical significance of novel biomarkers such as GRP and TORR in the context of acute critical illnesses requiring intensive care. GRP appeared to be a promising biomarker associated with calcium metabolism and tissue calcification, while TORR reflected oxidative stress status. Since oxidative stress is an integral component of inflammatory pathways, the simultaneous evaluation of GRP and TORR in our study provided novel insights into their interrelationships, which had not been extensively investigated in previous research.

By simultaneously assessing the effects of GRP and TORR on inflammation, oxidative stress, and calcium homeostasis, our study contributed new data to this emerging field. In particular, the negative correlation observed between GRP and serum calcium levels suggested that calcium metabolism might be influenced by inflammation and oxidative stress. Given the limited existing evidence on the association of these biomarkers with chronic inflammatory diseases, our study provided an important contribution to the current literature. Furthermore, considering that few studies had explored the relationship between GRP and TORR in chronic inflammatory conditions, our findings offered a novel perspective. The correlations identified between these biomarkers and various biochemical and hormonal parameters might yield valuable insights into the complex biology of inflammation and oxidative stress. Nevertheless, the clinical utility of GRP and TORR as stand-alone biomarkers remains limited without confirmation of their incremental value beyond traditional inflammatory markers and validation against clinical outcomes.

## Figures and Tables

**Table 1:** Comparison of Sociodemographic and Laboratory Parameters Between ICU Patients and Non-Critically Ill Controls.

Parameters	Control (n = 60)	Patients (n = 93)	*p*-Value
	**Mean ± SD, n (%)**		
Age (years)	59.2 ± 21.4	63.1 ± 20.0	0.190
Gender			0.260
- Male	42 (70%)	50 (53.8%)	
- Female	18 (30%)	43 (46.2%)	
Diabetes mellitus	5 (8.3%)	33 (35.5%)	<0.001
Hypertension	6 (10%)	39 (41.9%)	<0.001
Cardiovascular disease	7 (11.7%)	45 (48.4%)	<0.001
Chronic kidney disease (CKD)	3 (5%)	27 (29%)	<0.001
Respiratory system disease	2 (3.3%)	20 (21.5%)	<0.001
WBC (×10^3^/µL)	8.0 ± 4.7	12.6 ± 3.9	<0.0001
Neutrophils (×10^3^/µL)	4.5 ± 2.0	9.2 ± 3.8	<0.0001
Monocytes (×10^3^/µL)	0.5 ± 0.2	0.8 ± 0.4	0.010
Lymphocytes (×10^3^/µL)	2.5 ± 0.9	1.6 ± 0.7	0.020
MPV (fL)	8.5 ± 1.1	10.2 ± 1.3	0.030
Red blood cells (RBC, ×10^6^/µL)	4.8 ± 0.5	4.2 ± 0.6	0.040
Hemoglobin (Hgb, g/dL)	13.5 ± 1.2	11.3 ± 2.0	<0.010
Ferritin (ng/mL)	120 ± 70	540 ± 320	<0.001
Procalcitonin (PCT, ng/mL)	0.31 ± 0.41	4.46 ± 13.3	0.006
Calcium (Ca, mg/dL)	9.43 ± 0.4	7.89 ± 0.6	<0.0001
Gla-rich protein (GRP, ng/mL)	3.85 ± 0.68	4.19 ± 1.1	0.020
Immature granulocytes (IGs, ×10^9^/µL)	0.03 ± 0.01	0.12 ± 0.06	0.001
TORR (%)	116.5 ± 24.5	188.5 ± 180.9	0.030
CRP (mg/L)	5.7 ± 15.5	104.2 ± 84.0	<0.0001

**Table 2:** Correlation Analysis of GRP, TORR, and Biochemical Parameters in ICU Patients.

		WBC	Neut	Mon	Lym	MPV	Ferritin	PCT	Ca	GRP	IGs	TORR
WBC	r	1.00										
	p	1.00										
Neut	r	0.78	1.00									
	p	0.001	1.00									
Mon	r	0.06	−0.01	1.00								
	p	0.581	0.940	1.00								
Lym	r	0.03	−0.01	−0.27	1.00							
	p	0.769	0.913	0.015	1.00							
MPV	r	0.40	0.31	0.38	−0.18	1.00						
	p	0.001	0.003	0.001	0.020	1.00						
Ferritin	r	0.43	0.46	0.25	−0.21	−0.05	1.00					
	p	0.001	0.001	0.002	0.010	0.728	1.00					
PCT	r	−0.18	−0.19	−0.09	0.10	−0.03	−0.28	1.00				
	p	0.002	0.001	0.048	0.036	0.792	0.002	1.00				
Ca	r	0.54	0.58	0.32	0.25	0.27	−0.09	−0.35	1.00			
	p	0.001	0.001	0.002	0.014	0.002	0.061	0.001	1.00			
GRP	r	0.47	0.51	0.29	−0.31	0.25	0.30	0.34	−0.63	1.00		
	p	0.001	0.001	0.003	0.002	0.003	0.002	0.001	0.001	1.00		
IGs	r	0.48	0.44	0.27	−0.30	0.26	0.33	0.36	−0.65	−0.46	1.00	
	p	0.010	0.020	0.004	0.001	0.002	0.001	0.001	0.001	0.001	1.00	
TORR	r	0.48	0.44	0.27	−0.30	0.26	0.33	0.36	−0.65	−0.46	0.49	1.00
	p	0.010	0.020	0.004	0.001	0.002	0.001	0.001	0.001	0.001	0.010	1.00

Statistically significant difference. r : <0.2 Very weak correlation or no correlation. 0.2–0.4 Weak correlation. 0.4–0.6 Moderate correlation. 0.6–0.8 High correlation. >0.8 it is interpreted that there is a very high correlation.

**Table 3:** Linear regression analysis results for patients in the ICU.

Variable	B	St. Error	Beta	t	*p*-Value
GRP (ng/mL)	0.85	0.25	0.42	3.40	0.001
TORR (%)	0.12	0.05	0.33	2.45	0.015
CRP (mg/L)	0.09	0.03	0.30	3.00	0.003
WBC (×10^3^/µL)	0.40	0.14	0.28	2.86	0.005
IGs (×10^9^/µL)	2.60	0.90	0.29	2.89	0.006
PCT (ng/L)	0.07	0.02	0.26	2.70	0.008
Ca (mg/dL)	−0.65	0.18	−0.35	−3.61	0.001

**Table 4:** ROC analysis results for patients in the ICU.

Variable	AUC (95% CI)	Cut-Off	Sensitivity (%)	Specificity (%)	*p*-Value
GRP (ng/mL)	0.76 (0.68–0.84)	>4.0	72	70	0.001
TORR (%)	0.79 (0.71–0.86)	>160	75	73	0.001
CRP (mg/L)	0.88 (0.82–0.94)	>30.0	85	80	<0.001
WBC (×10^3^/µL)	0.81 (0.73–0.89)	>11.0	78	75	<0.001
IGs (×10^9^/µL)	0.77 (0.69–0.85)	>0.10	74	75	0.001
PCT (ng/L)	0.74 (0.65–0.83)	>0.5	70	68	0.004
Ca (mg/dL)	0.71 (0.62–0.80)	<8.2	65	70	0.008

## Data Availability

Data is available upon request to the corresponding author.
